# Development and Validation of a Universal Eating Monitor (UEM) for Distinguishing the Intake of Multiple Foods and Macronutrients

**DOI:** 10.3390/nu17182929

**Published:** 2025-09-11

**Authors:** Li Xue, Ying Liu, Huihui Mei, Ying Yu, Huanan Zhang, Lin Gao, Zengguang Jin, Lu Wang, Chaoqun Niu, John R. Speakman

**Affiliations:** 1 Shenzhen Key Laboratory of Metabolic Health, Center for Energy Metabolism and Reproduction, Shenzhen Institute of Advanced Technology, Chinese Academy of Sciences, Shenzhen 518055, China; 2 University of Chinese Academy of Sciences, Beijing 101408, China; 3 Key Laboratory of Molecular Pharmacology and Drug Evaluation, Ministry of Education, School of Pharmacy, Yantai University, Yantai 264005, China; 4 Institute of Genetics and Developmental Biology, Chinese Academy of Sciences, Beijing 100101, China; 5 School of Biological and Environmental Sciences, University of Aberdeen, Aberdeen AB24 2TZ, UK

**Keywords:** universal eating monitor (UEM), feeding table, energy intake, macronutrients, dietary microstructure, repeatability, positional effects

## Abstract

**Background/Objectives:** Dietary microstructure affects energy intake. Traditional Universal Eating Monitors (UEMs) offer accuracy but are limited for monitoring diverse diets. We developed the ‘Feeding Table’, a novel UEM that simultaneously tracks intake of up to 12 foods, enabling high-resolution monitoring of eating microstructure for multiple foods simultaneously. **Methods:** Forty-nine healthy volunteers participated: 15 (10 male, 8 female) in a location preference experiment and 31 (15 male, 16 female) in a standard meal test. The location preference study involved four weekly sessions. Participants received a standardized breakfast based on individual energy needs; lunch intake was measured 3 h later with food items in pseudo-randomized positions. The standard meal test occurred over two consecutive days to assess the Feeding Table’s performance in monitoring eating behavior under standardized conditions. **Results:** In two consecutive days of standard meal tests, the Feeding Table showed reasonable day-to-day repeatability for energy and macronutrient intake (energy: r = 0.82; fat: r = 0.86; carbohydrate: r = 0.86; protein: r = 0.58). Among the four repeated intake measurements, the results demonstrated high intra-class correlation coefficients (ICCs: energy 0.94, protein 0.90, fat 0.90, and carbohydrate 0.93). No significant positional bias was observed (energy: *p* = 0.07; macronutrients: *p* = 0.70). **Conclusions:** The Feeding Table maintains UEM accuracy while enabling multi-food, real-time monitoring of dietary microstructure and food choice, offering enhanced precision for studying eating behaviors.

## 1. Introduction

Accurate monitoring of food choice, preference, and intake is pivotal for understanding dietary impacts on health and metabolic processes. Currently, two primary approaches dominate the field: laboratory studies and naturalistic studies. Naturalistic studies, which include food frequency questionnaires and 24-h recalls [1] rely almost completely on self-reported information which is subject to memory issues and other biases. Recent data comparing intake records to doubly-labelled water suggest 27 to 38% of 24-h food intake recalls of adults, in large dietary surveys, are implausible [2]. Food diaries, including photo-diaries, overcome the memory issue, but still rely on individuals reporting in their diaries everything they eat, and impose a much greater burden on subjects. With the advance of artificial intelligence (AI), the widespread use of smartphones has significantly reduced the difficulty of dietary recording, and the continuous development of databases and algorithms has made their practical application increasingly feasible. Yet several limitations remain due to constraints such as photography methods, containers, and food image databases [3,4,5]. However, despite these advances, the accuracy of such methods remains an issue [4]. Laboratory studies, on the other hand, address the inherent limitations of naturalistic research by employing more controlled and direct methodologies. Common techniques involve pre- and post-meal weighing [1], as well as the use of specialized dietary monitoring devices such as a smart buffet setup with integrated weighing scales [6], food-intake gesture sensors [3], and a universal eating monitor [7] that tracks continuous eating behaviors [1]. These technologies provide a higher degree of precision in dietary intake data, but they sacrifice the normal setting of food intake, and may hence be less representative of intake during daily life.

The Universal Eating Monitor (UEM), developed by Kissileff [7], has become an indispensable tool for monitoring food intake in laboratory settings. This system features a custom-designed table integrated with scale(s) that can be either visibly embedded or concealed within the tabletop. It also includes advanced data recording systems and self-reporting mechanisms such as the Sequential Intake Pattern Method (SIPM) [8,9] and the Simultaneous Multiple Portion Method (SMPM) [10]. Beyond quantifying dietary energy and macronutrient intake, the UEM tracks eating behavior metrics like eating rate and changes in cumulative food intake [8,11,12]. This comprehensive approach provides a richer, more detailed record of food intake, enhancing the depth and accuracy of nutritional assessments.

Previous studies have evaluated the repeatability of the UEM [13,14], revealing its excellent stability in monitoring human eating behaviors. Key metrics include initial eating rate, changes in eating rate, eating duration, and meal size. However, some studies have reported lower correlation coefficients for eating rate changes when measurements were repeated one week apart. For instance, using yogurt resulted in a low correlation coefficient of 0.16 for eating rate between two repeated sessions [13]. Possible reasons include participants becoming more familiar with the process, leading to altered eating rates during the second session, and the inherent variability in consuming semi-solid foods like yogurt, which may be more susceptible to external and psychological factors compared with solid foods.

Similar findings were observed with ‘pudding’ [15], where the correlation coefficient for eating rate in consecutive sessions was higher, at 0.49, but still relatively low. A study involving four repeated UEM sessions spaced one week apart [13], using sandwiches as solid food, revealed low eating rate correlation coefficients of 0.20 between sessions for females, whereas the corresponding value for males was 0.64.

Despite the mature application of the UEM in food intake monitoring, existing research has predominantly focused on the intake of single-foods. This focus arises because traditional UEM setups typically incorporate only one scale, simplifying data analysis yet allowing robust quantification of participants’ hunger and satiety over time [9,16,17,18,19]. However, individuals rarely consume only one type of food with a fixed composition during meals. Moreover, the intake of a single food type offers no insights into food preference or macronutrient intake patterns during a single meal. Several National Dietary Guidelines [20,21,22] emphasize that diverse food sources provide more comprehensive nutrition, which is beneficial for health. Environmental factors, genetics, and lifestyle further contribute to the complexity of dietary structure. Specifically, the distribution of Macronutrients that supply energy is related to endocrine diseases such as obesity [23], diabetes [24], and hypertension [23].

In dietary intake monitoring research, buffet-style monitoring is commonly adopted in laboratory settings for studies involving multiple food combinations, aiming to enhance the authenticity of dietary choices. However, such methods are generally unable to achieve precise temporal resolution of food intake. One study developed an AI-powered smart dining table system that identifies food and estimates intake by analyzing grayscale images. Yet, its practical application remains limited by challenges such as food occlusion errors and high dependency on experimental conditions like lighting and camera angles, making widespread adoption difficult in the near term [3]. Meanwhile, emerging wearable sensing technologies offer more feasible solutions for monitoring the microstructure of eating behaviors, primarily categorized into chemical biosensors and physical biosensors [25,26,27]. These two types are functionally complementary and can work synergistically to enable more comprehensive dietary monitoring. These sensors are typically worn on areas such as the face [25,28,29], teeth [30,31], and fingers [32], and capture partial indicators of dietary microstructure by monitoring facial muscle activity [28,29], food weight [32], and integrating camera-based detection of chewing and swallowing motions [28]. The advancement of these AI-driven technologies holds promise for achieving comprehensive, continuous, and non-invasive monitoring of eating behavior.

Another approach involved placing two meals on separate scales to record the weight of food consumed by each participant [33]. Although similar in appearance to UEM design, this method did not include measured changes in eating rates because the location where the food was selected was separate from the location where it was consumed, and hence this approach essentially falls under pre- and post-meal weighing methods. In a buffet monitoring experiment, volunteers were provided with 12 types of food to assess the repeatability of buffet monitoring and the impact of hunger on eating behavior [33]. Video recordings were used to count bites, but no quantitative analysis was performed on actual intakes on a fine timescale.

Currently, monitoring eating behaviors involving multiple foods primarily relies on pre- and post-meal weighing, limiting researchers to qualitative assessments through video monitoring or image AI grayscale recognition, rather than quantitative monitoring. Therefore, there is a need to improve Universal Eating Monitor (UEM) technology to achieve more precise and comprehensive dietary monitoring of intake of multiple foods presented simultaneously. Additionally, while the UEM demonstrates excellent repeatability in single-food studies, further validation is required for the situation when multiple foods are available. This highlights the necessity of developing a system capable of simultaneously and independently monitoring multiple foods and macronutrient intake rates. Here we describe such equipment, named Feeding Table, able to significantly enhance our understanding of eating behaviors and their health impacts, providing robust support for personalized nutritional advice and the prevention of diet-related diseases.

## 2. Methods

### 2.1. The Structure of Feeding Table

**Figure 1:**
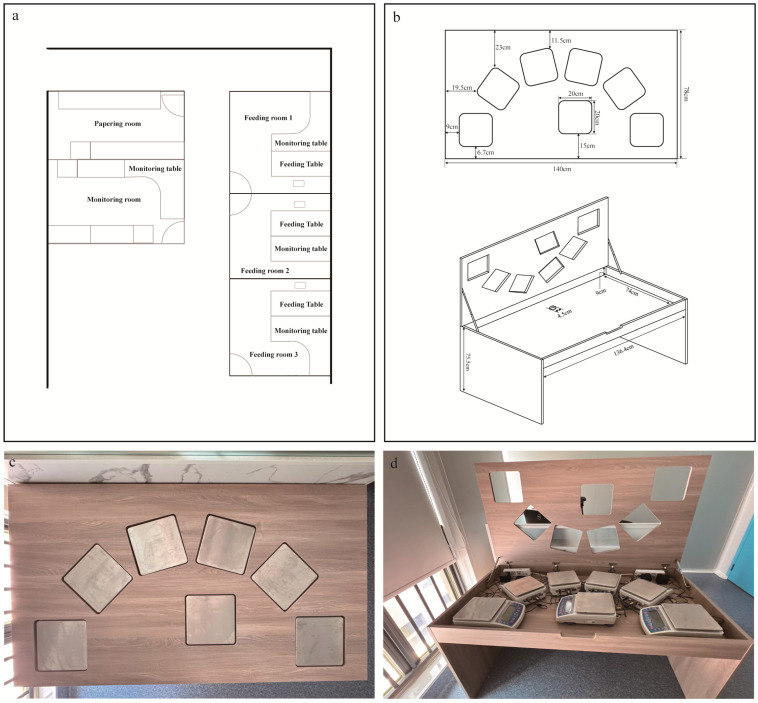
(**a**) Schematic floor plan of feeding rooms specialized in the assessment of dietary energy intake. **Top-left**: Food Preparation Area, used for the preparation, weighing, and heating of test meals. **Bottom-left**: Dietary Intake Monitoring Room, where staff monitor food consumption in the testing room, facilitate the replacement of dishes, and observe real-time data recording. **Right**: Dietary Intake Testing Room, which contains a food table and monitoring computers for recording data. (**b**) Feeding Table top design diagram (140 cm × 78 cm × 75.5 cm). **Upper**: a top-down view of the Feeding Table, featuring 7 designated balance placement points. Among these, 6 balances (A, B, C, D, E, and F) are arranged in an axially symmetric pattern, with each positioned at an equal distance from the center point along the long edge of the table. The seventh balance (G) is reserved for condiments (if required). **Bottom**: an internal view of the food table (136.4 cm × 74 cm × 9 cm), designed to accommodate balances used for weighing foods. (**c**) Top-down view photograph of the Feeding Table. (**d**) Photograph of the internal structure of the Feeding Table.

We expanded the single balance UEM system by building a ‘Feeding Table’ which included five balances, all with large top pan areas, capable of monitoring up to 12 different foods simultaneously, by having multiple dishes on each balance. Data are collected every 2 s and transmitted in real time to a computer via the balances’ built-in software for recording. In the feeding room, there is a custom-made solid wood Feeding Table (Figure 1). In front of the table is a partition (140 cm × 35 cm) topped with a standard video camera to record the participant’s eating process and also identify which food was taken from each balance. Behind this partition is a desk equipped with a computer connected to the balances continuously recording the balance outputs, and a thermal imaging camera trained on the subject to give forehead temperature (for studies of the impact of subject body temperature on intake) (Figure 1). For operational convenience, the computer in the Feeding Room is remotely controlled by a computer in the adjacent Monitoring Room via ‘Sunlogin’, (a service software for remotely managing and controlling computers: Oray, Shanghai). This setup allows researchers to observe the experiment in real time from the Monitoring Room without disturbing the subject. This constant monitoring also allows the researcher to intervene to prevent data loss due to accidental contact with the table, and replenish any food item that is getting close to being completely consumed. In our system we have three feeding rooms, each with a Feeding Table, that are all monitored from the single monitoring room.

The solid wood dining table features a hinged panel that can be opened and a balance compartment of dimensions: 140 cm × 78 cm × 75.5 cm (Figure 1). To facilitate opening and closing the balance compartment, the panel and the side of the compartment are connected by springs, which provide a cushioning effect to prevent accidents such as pinching or crushing. A small opening at the end of the balance compartment, away from the participant, allows for connections between the scales and the computer. The panel has five square holes arranged along an arc centered on its midpoint, each hole measuring 20 cm × 20 cm × 1.8 cm, for placing balances. Previous work has shown that consumption and preference of particular foods can be affected by adding flavorings to the food. We therefore wanted to separate intake driven by the macronutrient and energy contents from intake driven by added flavorings. A sixth hole of the same size is also provided near the participant’s side. This balance had a set of condiments in small bowls on it that individuals could dip into any food they had already selected from the other food bowls. The goal of separating condiments from the main food items was to enable a clear distinction between macronutrients and energy derived from the food, versus consumption driven by flavor derived from the condiments.

We performed two experiments to explore position preference and repeatability of the intake measurements using the Feeding Table.

### 2.2. Position Preference Experiment

#### 2.2.1. Participants

In the position preference experiment, we recruited and screened healthy volunteers aged ≥ 18 years via a questionnaire distributed on social media (WeChat). Exclusion criteria included: females who were planning to conceive, pregnant, or breastfeeding; individuals with dietary restrictions due to religious beliefs; those who had lost more than 2.5 kg in the past three months; participants who had undergone surgery in the past six months; individuals with irregular lifestyles or plans to lose weight within the next month; and those with endocrine disorders, cardiovascular diseases, infectious diseases, gastrointestinal illnesses, or psychiatric conditions. A total of 20 volunteers were initially recruited, but only 15 completed the experiment. The reasons for not completing the experiment included dysmenorrhea, gastrointestinal discomfort, influenza infections, unanticipated dietary alterations, and time limitations. The basic information of the volunteers who completed the experiment are shown in Table 1.

#### 2.2.2. Procedure

Participants were informed that this was a pilot study aimed at selecting palatable foods for subsequent experiments. They were instructed to fast overnight and arrive at the laboratory in a fasting state (without consuming water) according to the scheduled time. Anthropometric measurements included height and fasting body weight and were assessed using a TANITA Bioelectrical Impedance Analyzer (BIA). Breakfast was designed to provide energy equivalent to the estimated EE over three hours. After breakfast, participants were free to engage in their usual daily activities but were prohibited from consuming any calorie-containing foods, taking medications or supplements, and engaging in moderate-to-high-intensity physical activity; they could drink ambient temperature water. Lunch was provided on the Feeding Table. Ten minutes before lunch, participants completed the Three-Factor Eating Questionnaire (TFEQ-R21) and the Visual Analogue Scale (VAS) to assess emotional eating, pre-lunch hunger, and fullness. Immediately following lunch, participants filled out post-meal questionnaires and VAS to evaluate their liking of the provided meal and post-meal hunger and fullness. The experiment was repeated four times, with body weight updated before each breakfast session. The interval between repeated sessions was one week.

#### 2.2.3. Breakfast

In the position preference experiment we provided participants with breakfast, consisting of steamed buns (energy: 422.6 kcal/100 g, macronutrient energy distribution: 84% carbohydrate, 12% protein, and 4% fat) and unlimited ambient temperature drinking water (25 °C). The formula to calculate how much food to provide each subject relative to energy requirements was as follows:

$$Weight\left(g\right)=\;\frac{TEE(kcal/h)\times Time(h)}{Energy\_density\times Edible\_ratio\times Digestibility}$$

The total energy expenditure (*TEE*) was estimated based on a formula from previous laboratory research findings [2]; the food’s energy density was measured using a Bomb calorimeter; the time was the interval between breakfast and lunch (3 h); the edible portion is the ratio of dry weight to fresh weight, determined by weighing before and after drying in an oven at 65 °C for four weeks; and the estimation of digestibility was referenced from the research by energy value of foods [34]. The breakfast food weight calculation code is provided in Supplementary File S2. Participants were required to finish their breakfast within 15 min to standardize as much as possible downstream hormonal effects [35].

#### 2.2.4. Lunch—Using the Feeding Table

The lunch consisted of four food items: steamed rice, braised pork trotters, green beans with lean pork, and green vegetables. Detailed information on the energy content and macronutrient composition of the selected foods is provided in Table 2. Additionally, participants were provided with ambient temperature Coca-Cola and drinking water. The goal of using a simplified food set was to investigate the impact of food placement on dietary intake. To avoid order effects, the positioning of food items during each repeated session was determined using a randomly generated Latin square matrix, ensuring that each participant experienced a randomized and counterbalanced arrangement of food placements.

During the lunch, participants were informed about the presence of the Feeding Table to prevent data anomalies or data loss due to contact with the table surface. Participants were instructed to eat until they felt strongly satiated. When three-quarters of any dish was consumed, it was replaced with a fresh one. Unlimited supplies of cola and drinking water were available.

### 2.3. Repeatability Test

#### 2.3.1. Procedure

**Figure 2:**
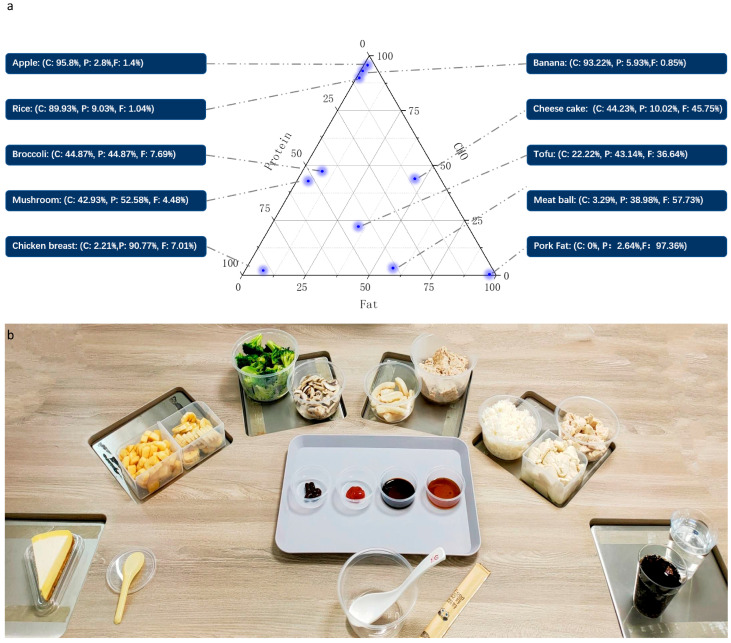
Ternary plot of macronutrient distribution in the standard meal. (**a**). Picture of standard testing meal, the types of food on the scale are fixed, but their order is randomized by a script. (**b**). Macronutrient ternary phase distribution of standard test meals.

In this test, we applied the same recruitment criteria. A total of 31 volunteers were recruited, 28 participants completing the experiment. The repeatability of the test meal intake was assessed during lunch sessions conducted on two consecutive days. The tests were carried out after participants had followed their own habitual breakfast routines and fasted for 4 h. A set of food items (n = 10) were placed in fixed positions (Figure 2). Prior to the test, participants were also informed about the presence of the Feeding Table and allowed to familiarize themselves with the laboratory setup and the food items provided. This was done to minimize data anomalies or loss that could result from food touching the table surface, or participants’ novelty response to the experimental environment. Participants were instructed to eat until they felt strongly satiated. Throughout the experiment, the eating process and food intake were recorded in real time using the Feeding Table. When approximately three-quarters of any dish was consumed (as judged from the monitoring room), it was replaced with a fresh one.

#### 2.3.2. Standardized Test Meals

To select foods to place on the table, we referred to the first and second volumes of the 6th edition of the “China Food Composition Table” and selected foods based on the following criteria: foods without regional characteristics, mild flavor, moderate price, and representative of common Chinese dietary types. To ensure comprehensive coverage of major macronutrient proportions, we chose foods with extreme macronutrient content, such as white rice with over 80% carbohydrates, chicken breast with over 80% protein, and pork fat with over 80% fat. We added to these, three types of foods where one macronutrient was adjusted while the other two remained similar in proportion: broccoli (low carbohydrate, high fiber), mushrooms (low fat, moderate protein), a meat patty composed of equal parts lean and fatty meat, cheesecake (high fat, moderate carbohydrate and fat). We also added tofu, a food with balanced macronutrient composition, whose macronutrient profile is centrally located within the ternary plot. We also included fruits (apples and bananas) and beverages (water and cola) in the menu to mimic the daily diet of Chinese individuals. Using this combination of 10 foods and two beverages ensured broad coverage of macronutrient ratios. Detailed information on the energy content and macronutrient composition of the provided foods is in Figure 2 and Table 3.

### 2.4. Statistical Analysis

All statistical analyses were performed using GraphPad Prism 8, IBM SPSS Statistics 26, Origin 2018. Two-way repeated-measures ANOVA was conducted to examine the interaction effects of experimental session, balance unit, food type and intake, as well as macronutrient type and consumption. For variables such as fiber intake, meal duration, total energy intake, and pre- and post-meal hunger ratings, a one-way repeated-measures ANOVA was performed to assess within-subject variability across sessions and units. All ANOVA models included Bonferroni correction for multiple comparisons to control family-wise error rates. Assumptions of normality and sphericity were evaluated using the Shapiro–Wilk test and Mauchly’s test, respectively, and Greenhouse–Geisser corrections were applied when sphericity was violated. Pearson correlation coefficients and intra-class correlation coefficients (ICC) were calculated to evaluate the associations between energy intake, hunger ratings, macronutrient consumption, and experimental conditions (i.e., session and balance unit). Agreement in energy expenditure measurements across experimental sessions and balance units was assessed using the Bland–Altman method. The microstructural feeding behavior model was fitted using both LODE and a quadratic model, with model differences evaluated via *t*-tests. Furthermore, the effects of experimental session, macronutrient composition, and energy intake on initial eating rate, maximum intake, and the rate of decline in eating rate were analyzed using two-way repeated-measures ANOVA. All data were expressed as mean ± SEM.

### 2.5. The Usage of Generative Artificial Intelligence

The language editing, grammatical refinement, and textual optimization of this manuscript, as well as the code (Python 3.12.2) used for calculating parameters related to the LODE and quadratic models, were generated by Qwen, an artificial intelligence model developed by Alibaba Cloud. The code was implemented and executed using PyCharm Community Edition 2024.1. All scientific content, including study design, data interpretation, and methodological descriptions, was authored and verified by the researchers. AI tools were used only for language editing and code refinement.

## 3. Results

### 3.1. Macronutrient and Energy Intake Was Unaffected by Position of the Foods

**Figure 3:**
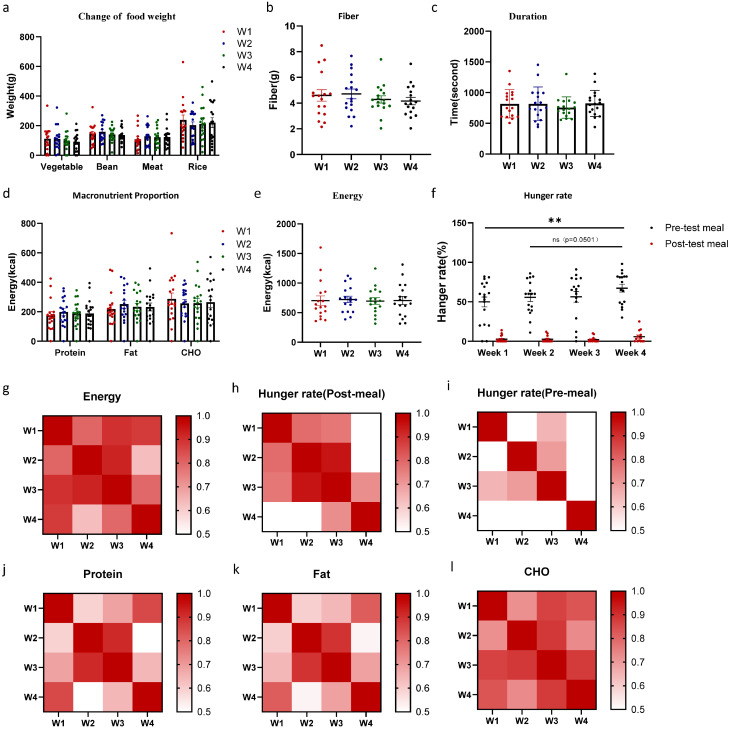
The Feeding Table demonstrated good repeatability in macro indicators and was unaffected by position bias. (**a**) The impact of trial times on the consumption of different dishes. (**b**) Variations in fiber intake across different trial times. (**c**) Variations in meal duration across different trial times. (**d**) The impact of macronutrients across different trial times. (**e**) Variations in energy intake across different trial times. (**f**) Variations in hanger rate across different trial times. Correlation analysis of total energy intake (**g**), post-meal hunger rate (**h**), pre-meal hunger rate (**i**), protein (**j**), fat (**k**), and CHO (**l**) have been shown. Data are presented as mean ± SEM and were analyzed by one-way ANOVA (**b**,**c**,**e**), and two-way repeated-measures ANOVA (**a**,**d**,**f**). ** *p* < 0.01.

In the position preference experiment, we first analyzed the consumption of identical dishes across four repeated trials where the location of the foods was randomized across the different balances on each trial (Figure 3). There was no interaction between food type and trial date on intake (Figure 3a, F (3, 60) = 0.12, *p* = 0.95). Regarding dietary fiber intake, no significant differences were found across trials (Figure 3b, one-way ANOVA, ± SEM, F (2.34, 36.64) = 1.49, *p* = 0.24). Meal duration did not differ across the four trials (Figure 3c) (F (2.48, 38.34) = 0.88, *p* = 0.44). Regarding energy intake and macronutrient intakes, neither of them differed significantly between trials (Figure 3d,e, Energy: F (1.58, 24.73) = 0.26, *p* = 0.72; Macronutrients: F (3, 68) = 0.03, *p* = 0.99). In the analysis of self-reported hunger, overall pre-meal hunger showed a significant difference (Figure 3f, F (3, 84) = 3.01, *p* = 0.03), with a notably significant higher level in week four when compared with week one (*p* = 0.002). Although the difference in pre-meal hunger between Week two and Week four did not reach statistical significance (*p* = 0.05), a rising trend was still observed in the self-reported ratings. In the correlation analysis, Intra-class Correlation Coefficient (ICCs, Table 4, energy: 0.94, protein: 0.90, fat: 0.90, CHO: 0.93) and Pearson(r) values for energy and macronutrient intake indicated good repeatability during four tests (Table 4). Overall, the macro-level dietary indicators monitored by the Feeding Table demonstrated good repeatability for multiple food items, particularly between the second and third repeated tests.

**Figure 4:**
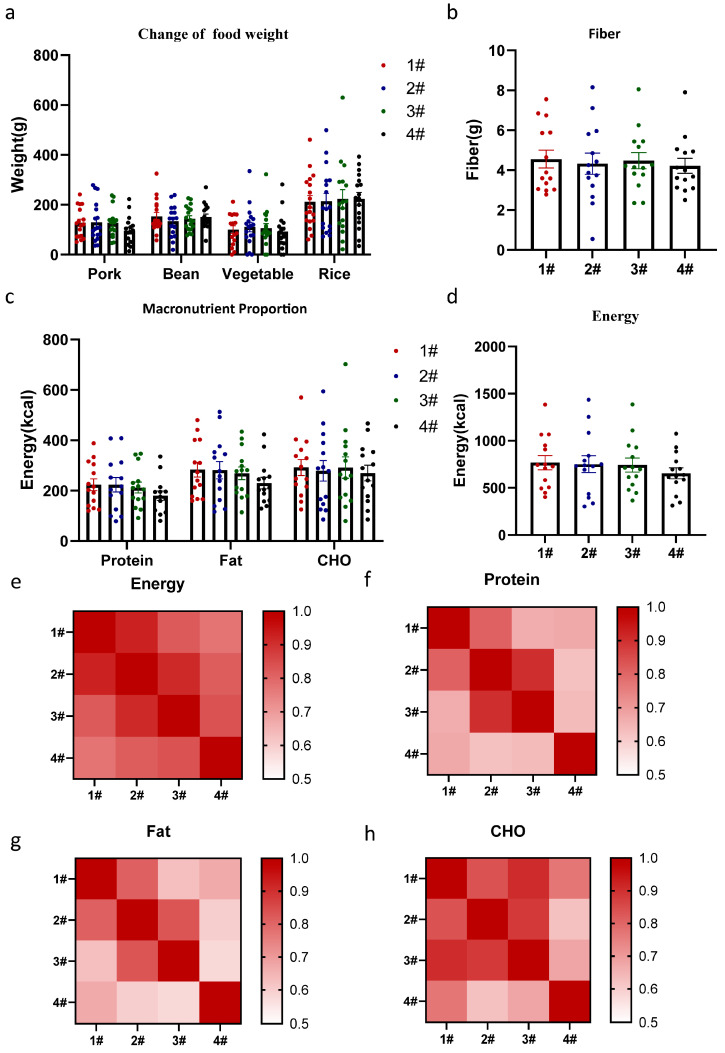
The Feeding Table was unaffected by position bias in macro indicators. (**a**) The impact of balance numbers on the consumption of different dishes. (**b**) Variations in fiber intake across different balance numbers. (**c**) The Impact of macronutrients across different balance numbers. (**d**) Variations in Energy intake across different balance numbers. Correlation analysis of total energy intake (**e**), protein (**f**), fat (**g**), and CHO (**h**) have been shown. Data are presented as mean ± SEM and were analyzed by one-way ANOVA (**b**,**d**), and two-way repeated-measures ANOVA (**a**,**c**). #: Balance number.

**Figure 5:**
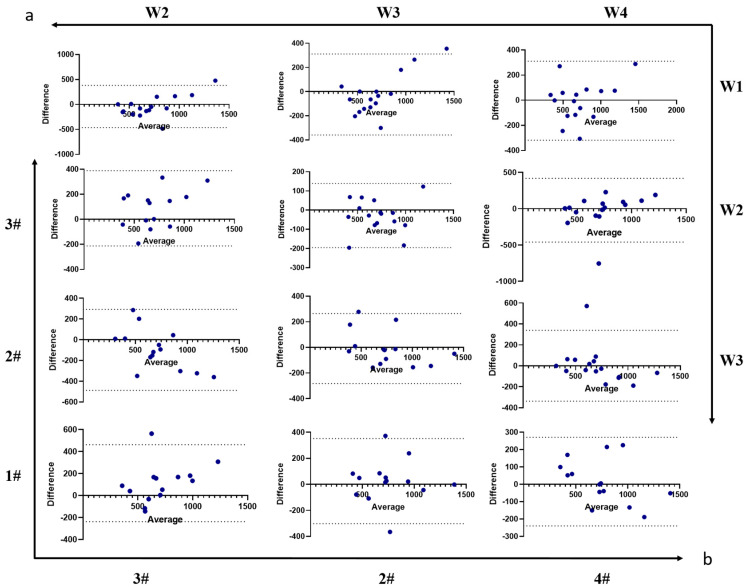
Bland–Altman analysis of energy intake from the location preference experiment. (**a**) Assessment of agreement in energy intake across varying trial repetitions. (**b**) Assessment of agreement in energy intake across varying trial repetitions (#: balance number).

In addition to the number of experimental sessions, variations in food intake weight on the different balances also warranted attention. By matching each participant with specific balance positions in the matrix, we analyzed differences in food intake when the same food appeared at the different balance positions (as shown in Figure 4). Balance positions did not significantly affect the weight of different foods consumed (Figure 4a, F (3, 68) = 0.12, *p* = 0.95). Similarly, no significant differences with balance location were found in fiber intake (Figure 4b, F (1.95, 25.33) = 0.35, *p* = 0.70), macronutrient energy ratios (Figure 4c, F (3, 52) = 0.47, *p* = 0.70), or energy intake (Figure 4d, F (2.39, 31.08) = 2.71, *p* = 0.07). In the correlation analysis, ICCs (Table 5, energy: 0.95, protein: 0.90, fat: 0.89, CHO: 0.93) and r values for energy and macronutrient intake indicated no significant differences when calculating the same food items placed at different balance positions. Furthermore, a Bland–Altman analysis was conducted to assess energy intake variability influenced by trial number (Figure 5a) and food position (Figure 5b). The results revealed mean differences (bias) of –19.1 kcal across the four trials and 22.4 kcal between balance units, with no evidence of proportional bias, indicating good agreement in energy intake assessment.

### 3.2. The Feeding Table Demonstrated Good Repeatability in Microstructure

**Figure 6:**
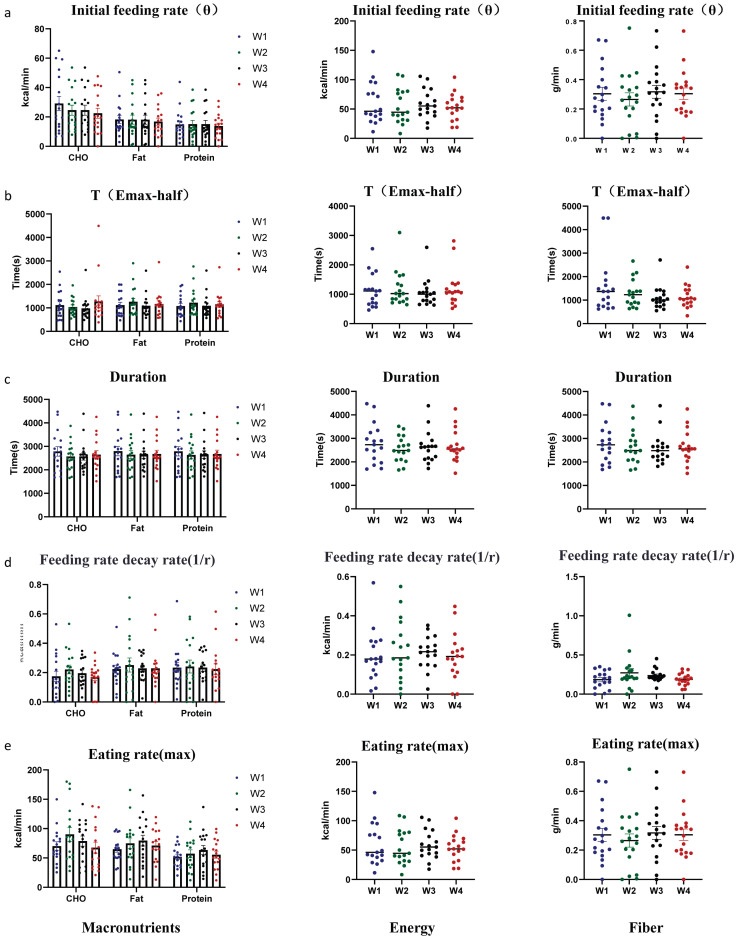
The food table demonstrated good repeatability in the measurement of feeding microstructure parameters (LODE model). (**a**) The effect of repetition count on the initial feeding rate. (**b**) The effect of repetition count on the time to reach half of the maximum intake. (**c**) The effect of repetition count on the duration. (**d**) The effect of repetition count on the feeding rate decay rate. (**e**) The effect of repetition count on the max eating rate. Data are presented as mean ± SEM and were analyzed by one-way ANOVA (middle: energy; right: fiber), and two-way repeated-measures ANOVA (left: macronutrients).

In our monitoring of eating microstructure at the Feeding Table, we compared a quadratic model with LODE model [10,11,12,36] for their ability to simulate the cumulative macronutrient, energy, and fiber intake curve. Although both models exhibit excellent fitting performance, the goodness-of-fit of the LODE model was significantly higher than that of the quadratic model (R^2^: Quadratic vs. LODE model 0.97 vs. 0.98, *p* < 0.0001) (Table 6). Additionally, using the LODE model, we calculated key parameters such as the initial eating rate (θ, Figure 6a), the time to reach half of the maximum intake (T(Emax-half), Figure 6b), the duration (Figure 6c), the feeding rate decay rate (1/r, Figure 6d), and the max eating rate (Figure 6e). These parameters showed good repeatability across the four repeated meals involving position changes.

### 3.3. Standard Meal Repeatability

**Figure 7:**
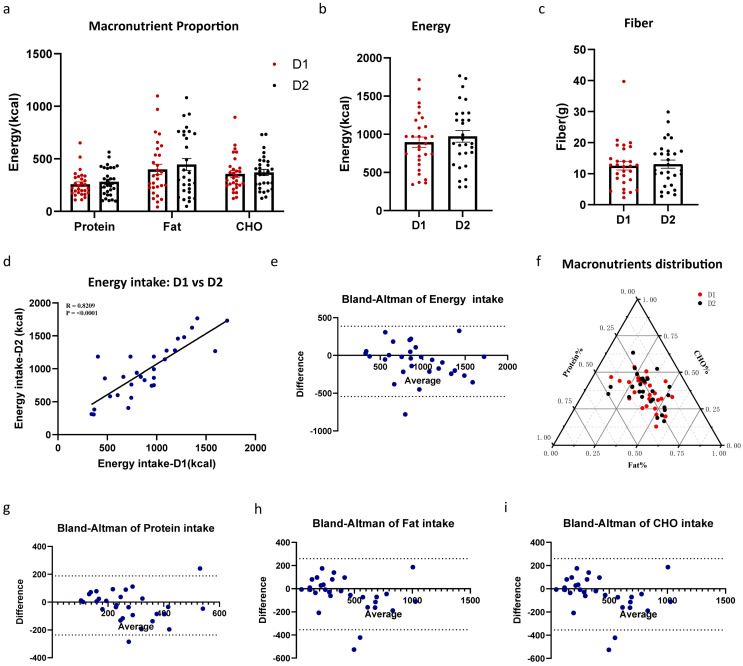
The standard meal demonstrated excellent repeatability in the Feeding Table’s application. (**a**) The impact of repeated experiments on the following day on the energy contribution ratios of macronutrients. (**b**) The impact of repeated experiments on the following day on the energy intake. (**c**) The impact of repeated experiments on the following day on the fiber intake. (**d**) Linear regression of energy intake D1 vs energy intake D2. The Bland–Altman analysis was conducted to compare energy intake (**e**), protein (**g**), fat (**h**), and carbohydrate (**i**) between Day 1 (D1) and Day 2 (D2). (**f**) Ternary plot of macronutrient distribution between Day 1 (D1) and Day 2 (D2). Data are presented as mean ± SEM and were analyzed by *t*-test (**b**,**c**), and two-way repeated-measures ANOVA (**a**).

To assess repeatability, we used the full Feeding Table with all 10 foods and 2 beverages and compared intakes across two consecutive days (Figure 7). The macronutrient energy ratios did not exhibit significant differences (Figure 7a,e, *p* = 0.49). From the perspective of energy intake and fiber, there were no significant differences between the test meals on the two consecutive days (Figure 7b,c, energy *p* = 0.09, fiber: *p* = 0.65). In the correlation analysis (Table 7), both experiments showed good repeatability in terms of energy intake (r = 0.83, ICCs = 0.90), macronutrient intake (protein: r = 0.58, ICCs = 0.78; fat: r = 0.86, ICCs = 0.91; CHO: r = 0.86, ICCs = 0.92), and fiber intake (r = 0.65, ICCs = 0.79). Bland–Altman analysis results are presented in Figure 7e,g–i, as well as Table 7, and revealed small mean differences (i.e., biases) between the two repeated measurements, with values of –7.3 kcal for protein, –47.5 kcal for fat, –14.4 kcal for carbohydrate, –77.8 kcal for total energy intake, and –0.52 g for cellulose. No proportional bias was detected across the range of intake levels, indicating a high degree of consistency between the two experimental sessions in the assessment of energy and macronutrient consumption. Overall, the standardized test meal demonstrated good repeatability. Exact levels of repeatability deduced here can be used to inform power analyses for future studies of the same target population.

## 4. Discussion

In this study, the linear ordinary differential equations (LODE) model was applied to fit cumulative food intake curves, and the repeatability of dietary microstructure across repeated measurements was assessed. The results showed that key parameters derived from the LODE model—including initial eating rate, maximum intake (Emax), and the decay rate of eating rate (r)—did not differ significantly between sessions, indicating good within-individual stability and supporting their use as reliable behavioral phenotypes in longitudinal studies.

In the experiment evaluating positional preferences on the Feeding Table, we examined the repeatability and potential location bias in the intake of energy, macronutrients, and dietary fiber across the four balanced positions (1, 2, 3, and 4). No significant differences were observed, and overall repeatability was good. This suggests that all four positions can be used simultaneously to measure intake of multiple food items, thereby addressing a current limitation in UEM applications regarding the assessment of multi-food meals. However, it should be noted that factors such as the homogeneity and particle size of solid foods may influence the measurement of behavioral parameters like eating rate, and thus should be carefully controlled when selecting test foods.

Regarding model selection, the quadratic model remains the most commonly used analytical approach in UEM studies. In this model, the linear coefficient typically reflects the initial eating rate, while the quadratic coefficient represents changes in eating rate during the meal. A major limitation, however, is that the quadratic model may generate physiologically implausible predictions—such as a decline in cumulative intake—implying negative eating, which is not feasible in reality. In 2017, Thomas [12] introduced the LODE model, a first-principles-based approach, and demonstrated its superior performance in modeling cumulative eating curves compared to the quadratic model.

Due to limitations in the age range and health status of the participant population, the present findings primarily reflect dietary intake patterns in young, healthy individuals. Within this group, both the quadratic and LODE models provided good fits to energy and macronutrient intake data; however, the LODE model demonstrated significantly better goodness-of-fit, as indicated by higher R^2^ values and related metrics. From a parameter interpretability standpoint, the LODE model offers distinct advantages. The cumulative intake curve is theoretically S-shaped (though not universally so), and explicitly incorporates the initial phase of taste- and hunger-driven stimulation at meal onset. The model characterizes a rapid increase in eating rate at the beginning of the meal, followed by a gradual decline as satiation accumulates beyond a critical point. This dual-phase framework provides a more nuanced and biologically plausible representation of eating—a complex behavior regulated by multiple physiological and psychological factors—thereby enhancing our mechanistic understanding of ingestive behavior.

Moreover, the LODE model holds significant potential for interventional research, clinical studies, and therapeutic applications. Grounded in first principles, it does not require the inclusion of arbitrary “fudge factors” and is less dependent on high-temporal-resolution data (e.g., from UEM systems) [36], enabling its application to predict cumulative intake in non-UEM settings. In contrast, the quadratic model relies heavily on precise, continuous measurements and essentially serves as a retrospective description of actual intake. The LODE model, however, offers the prospect of prospective prediction—by adjusting key parameters (e.g., initial eating intensity, Emax, r) to simulate and potentially guide eating behavior. This predictive capacity could provide more timely and forward-looking tools for experimental parameter control, personalized intervention design, and real-time monitoring in clinical practice.

Looking ahead, future research should extend these analyses to more diverse populations encompassing different dietary patterns, age groups, cultural backgrounds, and health conditions. Such studies will provide robust empirical support for selecting the most appropriate modeling approach across contexts, further elucidate the regulatory mechanisms of eating behavior, and ultimately advance the fields of precision nutrition and behavioral intervention.

Nevertheless, the findings of this study are based on a small sample of young, healthy individuals who underwent strict screening, which limits the generalizability of the results. Therefore, the use of the Feeding Table to monitor eating behavior may not be representative of older adults, individuals with special dietary patterns, or those from different cultural backgrounds. Further work will be required in the future to characterize the responses and repeatability of measurements for these populations. Moreover, although the system captures more detailed data than traditional pre- and post-meal weighing methods, there are still several issues worth discussing and potential areas for improvement in future research.

### 4.1. Restriction 1: Awareness of the Monitoring or Not

Although the Feeding Table developed in this study enables simultaneous monitoring of multiple food items—more closely approximating participants’ real-world, mixed dietary patterns and thereby enhancing the simulation of natural eating behaviors—it remains inherently a laboratory-based system. Consequently, like other controlled laboratory methods, it faces the persistent trade-off between measurement accuracy and ecological validity.

In most studies using the Universal Eating Monitor (UEM), scales used to monitor food weight are concealed under tablecloths, and participants are not informed of this setup [9,37,38,39]. This design aims to enhance the ecological validity of the experiments, ensuring that participants’ natural eating behaviors are observed. Some studies argued that observations and measurements of eating behavior are only valid when participants are unaware they are being monitored, implying that awareness can alter behavior [1,7,13]. However, no empirical data were provided to support this claim. A study investigating the impact of hidden versus visible scales on participants’ food intake using a UEM found that, compared to the unaware group, the aware group did not differ in pasta consumption but significantly reduced their intake of cookies (*p* = 0.02) [8]. Notably, however, hiding the monitoring equipment led to a 59.6% loss of data, primarily because the scales touched the UEM table surface (53.2%) and, in some cases, potentially because volunteers became aware of the monitoring (6.3%). Data loss issues have been noted in several studies involving unaware participants, even though exact rates are not always specified.

Although it is often stated that placing a table cloth over the balances makes individuals unaware that they are being monitored, the UEM approach generally takes place in a laboratory setting, and individuals are often recruited knowing they are subjects in a feeding behavior experiment, because they are required by ethics to give informed consent. Hence, the extent to which they are truly unaware that they are being monitored is questionable. Completely covert observation of feeding behavior of individuals would increase the ecological validity of a study, but raises additional issues of privacy and ethical concerns regarding complete informed consent. We think this fundamental trade-off between accuracy and detail on one hand and ecological validity on the other is extremely difficult to resolve. It would be difficult to imagine setting up a feeding table system capable of the resolution in feeding behavior microstructure that we obtain in the laboratory, in a situation where ecological validity is preserved and individuals are unaware they are being measured. The use of wearable devices to monitor feeding structure is also affected by the issue that the individuals being monitored are aware they are wearing a device that is monitoring their behavior.

### 4.2. Restriction 2: Emptying Your Plate

When using the Universal Eating Monitor (UEM), it is important to be cautious of the “emptying your plate” effect [1,39]. Research has explored factors influencing normal food intake and found that the quantity of food provided can significantly impact how much participants eat. Survey results indicate that 61% of respondents tend to continue eating until their plate is empty [40], even after feeling full, possibly due to social conditioning against wasting food. In our actual monitoring sessions, the “emptying your plate” effect was frequently mentioned during post-meal interviews. Many volunteers reported finishing their food despite feeling sufficiently full when only a small amount remained on the plate. One approach to mitigate the “emptying your plate” effect is to provide unlimited food, as we did. However, this is not always successful as subjects who had finished a serving would sometimes decline additional food being added to the table to avoid leaving any uneaten, fearing they wouldn’t finish another full serving.

### 4.3. Restriction 3: Fast Overnight, Fixed Breakfast or Adherence to Lifestyle Habits

One critical consideration when using the Feeding Table is whether to provide participants with breakfast before the test meal, require them to fast overnight, or allow them to follow their usual eating habits. Currently, there is no standardized approach regarding breakfast provision during dietary intake testing. Some studies opt for not restricting breakfast consumption but require participants to maintain a 3–4 h fast before the meal [14,15,18,41]. Other studies mandate an overnight fast (exceeding 10 h), with [7,9,16,37,42] or without [6,13] providing a standardized breakfast on the day of the experiment. From an experimental design standpoint, allowing participants to follow their usual routines does not disrupt their daily habits but may overlook hormonal influences and introduce variability due to different nutrient intakes between individuals. For example, a high-fiber, high-protein breakfast can significantly reduce appetite and lower lunch intake [43]. Conversely, enforcing an overnight fast provides more controlled conditions but may ignore individual differences. Research indicates that compared to following their regular breakfast routine, overnight fasting leads to a significant increase in energy intake during the test meal, shifts the proportion of consumed carbohydrates and fats upwards, and decreases protein consumption [6]. However, from an energy balance perspective, this additional intake does not fully compensate for the skipped breakfast, leading to a decrease in total daily energy intake. In UEM studies, there is limited literature on how fasting state and breakfast consumption impact eating behavior microstructure. In our preliminary experimental results, we surveyed the daily breakfast habits of 112 volunteers [44], among whom 72 were habitual breakfast eaters and 40 were not. During lunch tests (standard testing meal) conducted according to their usual breakfast habits, no significant difference (*p* = 0.90) in energy intake was observed. Further research is needed to determine if standardizing fasting time before Feeding Table use is beneficial. It is clear, however, that in experiments involving diverse populations, fasting duration should be adjusted as necessary to prevent hypoglycemia and other potential risks.

## 5. Conclusions

In summary, we have developed the Feeding Table so that it is capable of simultaneously monitoring multiple foods, addressing the research gap in non-visual estimation of the microstructure of diverse food items. This Feeding Table demonstrates reasonable repeatability whether used with everyday foods or highly standardized test meals. Importantly, the placement of food items did not affect measurements of weight, energy intake, or macronutrient intake, ensuring reliable macro and micro indicators across different meals. This innovative tool offers a feasible, detailed, and ecologically valid method for monitoring dietary intake, enhancing the accuracy of dietary data. By providing more precise insights into eating behaviors, it will enable researchers and clinicians to better develop interventions that promote healthy eating habits and prevent diet-related diseases.

## Figures and Tables

**Table 1:** Basic subject characteristics for the two experiments on position preference and repeatability.

	Number	Age	Sex (M:F)	Height (cm)	Weight (kg)	BMI (kg/m^2^)	Handedness (R:L)
Position preference	18	29 ± 5.66 [21, 36]	10:8	168.45 ± 11.29 [148, 188.6]	66.37 ± 10.16 [49, 82.7]	24.67 ± 3.78 [18.22, 30.75]	14:4
Standard testing meal	31	26.26 ± 4.26 [22, 40]	15:16	168.60 ± 8.85 [154, 185]	59.41 ± 8.07 [45.4, 75]	20.81 ± 1.61 [18.07, 23.55]	31:0

**Table 2:** Composition of the four foods used in the position preference test.

Food (n = 4)	Protein (g/100 g)	Fat (g/100 g)	CHO (g/100 g)	Fiber (g/100 g)	Energy (kJ)
Pork	28.4	15.4	0	0	992
Long bean	5.86	4.95	7.50	1.66	393
Green Vegetable	1.44	1.74	3.2	1.34	130
Rice	2.6	0.3	25.9	0.3	485

**Table 3:** Composition of the Test Meal.

Food (*n* = 12)	Protein (g/100 g)	Fat (g/100 g)	CHO (g/100 g)	Fiber (g/100 g)	Water (g/100 g)	Energy (kJ)
Pork (fat)	2.4	88.6	0	0	8.8	3319
Pork (lean and fat)	36.8	54.5	3.1	0.8	39.9	1959.5
Chicken breast	24.6	1.9	0.6	0	71.7	499
Tofu	6.6	5.3	3.4	—	83.8	3512
Rice	2.6	0.3	25.9	0.3	70.9	486
Broccoli	3.5	0.6	3.7	1.6	91.6	111
Mushroom	38.7	3.3	31.6	17.2	9.2	1162
Apple	0.4	0.2	13.7	1.7	86.1	227
Banana	1.4	0.2	22	1.2	75.8	389
Cheese cake	5.3	24.2	23.4	—	—	1384
Butter	1.4	98	0	0	0.5	3715
Water	0	0	0	0	100	0
Coke	0	0	10.6	0	—	180

**Table 4:** ICC and correlation (r) values for energy and macronutrient intake based on 4 repetitions.

Food	Protein	Fat	CHO	Energy
ICCs	0.90 ^a^	0.90 ^a^	0.93 ^a^	0.94 ^a^
R value across 4 sessions				
W1 vs. W2	0.59	0.60	0.72	0.88
W1 vs. W3	0.69	0.65	0.87	0.67
W1 vs. W4	0.69	0.82	0.83	0.84
W2 vs. W3	0.91	0.89	0.88	0.83
W2 vs. W4	0.49	0.52	0.73	0.74
W3 vs. W4	0.64	0.69	0.88	0.73

One-way ANOVA, ±SEM, ^a^: *p* < 0.001.

**Table 5:** ICCs and correlation (r) for energy and macronutrients at different positions.

Food	Protein	Fat	CHO	Energy
ICCs	0.90 ^a^	0.89 ^a^	0.93 ^a^	0.95 ^a^
R value across 4 sessions				
1# vs. 2#	0.81	0.81	0.84	0.92
1# vs. 3#	0.66	0.63	0.91	0.82
1# vs. 4#	0.67	0.67	0.77	0.77
2# vs. 3#	0.90	0.83	0.88	0.91
2# vs. 4#	0.62	0.60	0.62	0.82
3# vs. 4#	0.63	0.58	0.68	0.84

One-way ANOVA, ±SEM, ^a^: *p* < 0.001. # balance number.

**Table 6:** Comparison of the goodness-of-fit between the Quadratic Model and LODE Model.

	Quadratic Model		LODE Model	
	R^2^	SD	R^2^	SD
Energy	0.97	0.03	0.99 ^a^	0.01
Protein	0.97	0.02	0.99 ^a^	0.01
Fat	0.97	0.03	0.98 ^a^	0.01
CHO	0.97	0.03	0.98 ^a^	0.02
Fiber	0.97	0.03	0.98 ^a^	0.01

*t*-test, ^a^: *p* < 0.0001.

**Table 7:** Correlation analysis of macronutrients in the Standard Test Meal.

	Correlation			Bland–Altman		
	r	*p*	95%CI	Bias	SD of Bias	95%CI
Protein	0.58	0.001	0.27 to 0.78	−7.26	126.7	−255.6 to 241.1
Fat	0.86	<0.0001	0.71 to 0.93	−47.54	156.6	−354.4 to 259.3
CHO	0.86	<0.0001	0.71 to 0.93	−14.39	90.29	−191.4 to 162.6
Energy	0.83	<0.0001	0.65 to 0.91	−77.82	237.3	−542.9 to 387.3
Fiber	0.65	0.0001	0.37 to 0.82	−0.52	6.123	−12.52 to 11.48

## Data Availability

The published article and Supplementary Information include the data used to generate the figures in the paper (Supplementary Data S1). The published article and Supplementary Information include the script used to calculate or analyze the data in the paper (Supplementary Data S2).

## Supplementary Materials

The following supporting information can be downloaded at: https://www.mdpi.com/article/10.3390/nu17182929/s1.
